# Highly conserved molecular pathways, including Wnt signaling, promote functional recovery from spinal cord injury in lampreys

**DOI:** 10.1038/s41598-017-18757-1

**Published:** 2018-01-15

**Authors:** Paige E. Herman, Angelos Papatheodorou, Stephanie A. Bryant, Courtney K. M. Waterbury, Joseph R. Herdy, Anthony A. Arcese, Joseph D. Buxbaum, Jeramiah J. Smith, Jennifer R. Morgan, Ona Bloom

**Affiliations:** 1 The Feinstein Institute for Medical Research, Center for Autoimmune and Musculoskeletal Disease, Manhasset, NY 11030 USA; 2 University of Kentucky, Department of Biology, Lexington, KY 40506 USA; 3 Icahn School of Medicine at Mount Sinai, Department of Psychiatry, New York, NY 10029 USA; 4 Marine Biological Laboratory, The Eugene Bell Center for Regenerative Biology and Tissue Engineering, Woods Hole, MA 02543 USA

## Abstract

In mammals, spinal cord injury (SCI) leads to dramatic losses in neurons and synaptic connections, and consequently function. Unlike mammals, lampreys are vertebrates that undergo spontaneous regeneration and achieve functional recovery after SCI. Therefore our goal was to determine the complete transcriptional responses that occur after SCI in lampreys and to identify deeply conserved pathways that promote regeneration. We performed RNA-Seq on lamprey spinal cord and brain throughout the course of functional recovery. We describe complex transcriptional responses in the injured spinal cord, and somewhat surprisingly, also in the brain. Transcriptional responses to SCI in lampreys included transcription factor networks that promote peripheral nerve regeneration in mammals such as Atf3 and Jun. Furthermore, a number of highly conserved axon guidance, extracellular matrix, and proliferation genes were also differentially expressed after SCI in lampreys. Strikingly, ~3% of differentially expressed transcripts belonged to the Wnt pathways. These included members of the Wnt and Frizzled gene families, and genes involved in downstream signaling. Pharmacological inhibition of Wnt signaling inhibited functional recovery, confirming a critical role for this pathway. These data indicate that molecular signals present in mammals are also involved in regeneration in lampreys, supporting translational relevance of the model.

## Introduction

A fundamental question in regenerative biology is why some organisms can regenerate their central nervous system (CNS), while others cannot^1^. Unlike mammals, lampreys, fishes, amphibians, and reptiles exhibit robust spontaneous regeneration and functional recovery after SCI^2–4^. The mammalian peripheral nervous system (PNS) also regenerates after injury^5^, and intraspinal neurons in mammals can do so when provided a permissive environment, such as a peripheral nerve bridge or growth factors^6–9^. However, the conserved molecular pathways that promote successful regeneration are unclear^10^.

We therefore set out to identify deeply conserved pro-regenerative pathways by determining the gene expression changes that occur after SCI in lampreys. The lamprey is a member of an ancient vertebrate lineage that diverged from a common ancestor of humans ~550 million years ago^11–13^. Despite this evolutionary distance, recent sequencing of the lamprey genome revealed molecular pathways that are conserved with mammals, including genes related to axon guidance and regeneration, synaptic transmission, neural patterning and neurodegeneration^12^. The organization of the lamprey CNS is highly analogous to human and other jawed vertebrates^14,15^. Remarkably, lampreys recover locomotor function (*e.g*. swimming) within 12 weeks after a complete spinal cord transection, which is supported by repair of the spinal lesion, axon regeneration, and synapse formation^16–20^ (Video S1). To better understand the molecular pathways supporting successful functional recovery, we used RNA-Seq to determine transcriptional profiles in spinal cord of lampreys after SCI. Because many of the regenerating descending axons extend from cell bodies located in the brain, we also profiled the supraspinal transcriptional responses in order to determine their contributions to the recovery process. We identified complex transcriptional responses in both spinal cord and brain throughout the 12 weeks after injury, including a number of expression changes mapping to the Wnt pathway, and used pharmacological blockade of Wnt signaling to demonstrate its critical importance in functional recovery.

## Results

### Spinal Cord Regeneration in Lampreys

**Figure 1:**
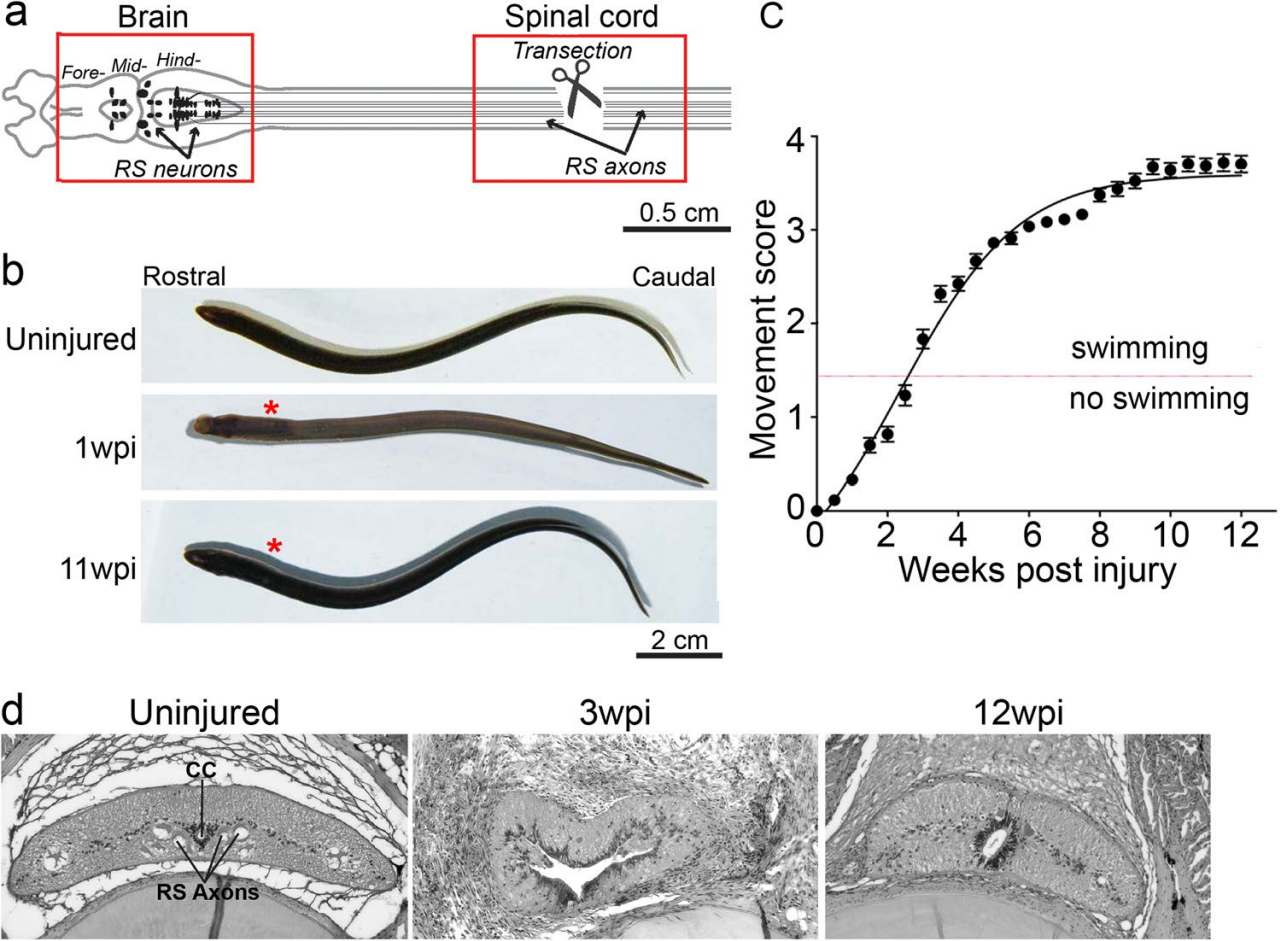
Robust functional recovery after complete spinal cord transection in lampreys. (**a**) Diagram of lamprey brain and spinal cord showing location of transection site and tissues collected for molecular profiling (red boxes). Somata of RS neurons (arrows) reside in the midbrain and hindbrain, and their axons (black lines) extend the length of the spinal cord. (**b**) Images of uninjured lampreys and at 1wpi and 11wpi. Asterisks indicate lesion site. (**c**) Quantitative scale of functional recovery (i.e. swimming): Movement scores are as described fully in^18^: 0-paralysis); 1-head wiggle only; 2-brief, abnormal swimming; 3-persistent swimming with abnormal body shape; 4-apparently normal swimming. Data points represent mean ± SEM (n = 27–66 animals per data point). (**d**) Histological sections of lamprey spinal cord from uninjured, 3wpi, and 12wpi demonstrating cytological changes during the recovery from SCI. CC = central canal, RS axons = reticulospinal axons.

The lamprey CNS contains many homologous structures that are shared with mammals, including a tripartite brain and spinal cord with motor and sensory circuits (Fig. 1a)^14,21–23^. Within the spinal cord are ~1200 reticulospinal (RS) axons, originating from somata in the midbrain and hindbrain, comprising the major descending pathway for initiating locomotion (Fig. 1a)^24^. Spinal cord transection severs all axons, leading to paralysis below the lesion that typically lasts 1–2 weeks (Fig. 1a,b; Video S1)^16–20^. Over the next 10–12 weeks post injury (wpi), lampreys spontaneously recover nearly normal swimming behaviors, which can be described via a quantitative scoring system, where 1 corresponds to an ability to curve into a “C” or “S” shape without translation to forward motion, 2 corresponds to an ability to achieve brief abnormal swimming, 3 corresponds to persistent but abnormal swimming and 4 is normal swimming. (Fig. 1b,c; Video S1) (n = 25–66 animals per time point; R^2^ = 0.97; t_1/2_ = 3.43 ± 0.12 weeks)^18,25^. Animals that reach stage 4 are considered to have achieved full functional recovery. This functional recovery is supported by lesion repair, regeneration of descending and ascending axons, and proliferation of cells that give rise to new neurons and glia^16,18,20,26–29^. Figure 1d shows the basic cytoarchitecture of the spinal cord during recovery from SCI, as initially shown by Rovainen^16^. Previously, it has been shown that ~50% of RS axons regenerate beyond the lesion and form synapses with appropriate postsynaptic targets^17,18^. In the brain, SCI triggers cell death and also regeneration of subpopulations of neurons with descending axons, as well as neurite sprouting from uninjured neurons^30–32^.

### Differential Expression and Enrichment Analysis of Molecular Pathways during Spinal Cord Regeneration and Functional Recovery in Lamprey

Uninjured spinal cord tissues contain many neuronal subtypes (e.g. motoneurons, sensory neurons, interneurons) and glia (e.g. ependymal cells, microglia/macrophages). In addition, we know that injury induces ependymal cells and microglia/macrophages to accumulate at the lesion site^28,33–35^, though the relative proportions of these and other cell types at each post-injury time point are unknown. Brain tissue contained somata of axotomized neurons with descending axons, as well as other neurons and glia that were not injured directly, but that respond to the injured state. Cell proliferation and neurite sprouting have also been reported in the lamprey brain after SCI^28,32^. To obtain gene expression profiles accompanying successful spinal cord regeneration and functional recovery in lampreys, we performed RNA-Seq on cDNA libraries generated from spinal cord tissue (1 cm) surrounding the lesion site in controls and at ten time points after SCI, ranging from 6 hours to 12 weeks, and in parallel, from brains of the same animals (Fig. 1a; boxes). RNA-Seq reads were mapped to annotated gene models from the published lamprey genome assembly (https://genome.ucsc.edu/cgi-bin/hgGateway?db=petMar2)^12^. Standardized expression values for all gene models are reported separately for each time point in brain and spinal cord (see Supplementary Table S1). To confirm homology-based annotations for selected key genes within this study, we performed new BLAST searches for several annotated homologs of human genes that are typically associated with regeneration, proliferation and cell death in mammals. These genes shared ~40–96% amino acid identity and high similarity (50–99% positives, which also include features like charge conservation), with the closest (presumptively orthologous) vertebrate sequence (see Supplementary Table S2).

**Figure 2:**
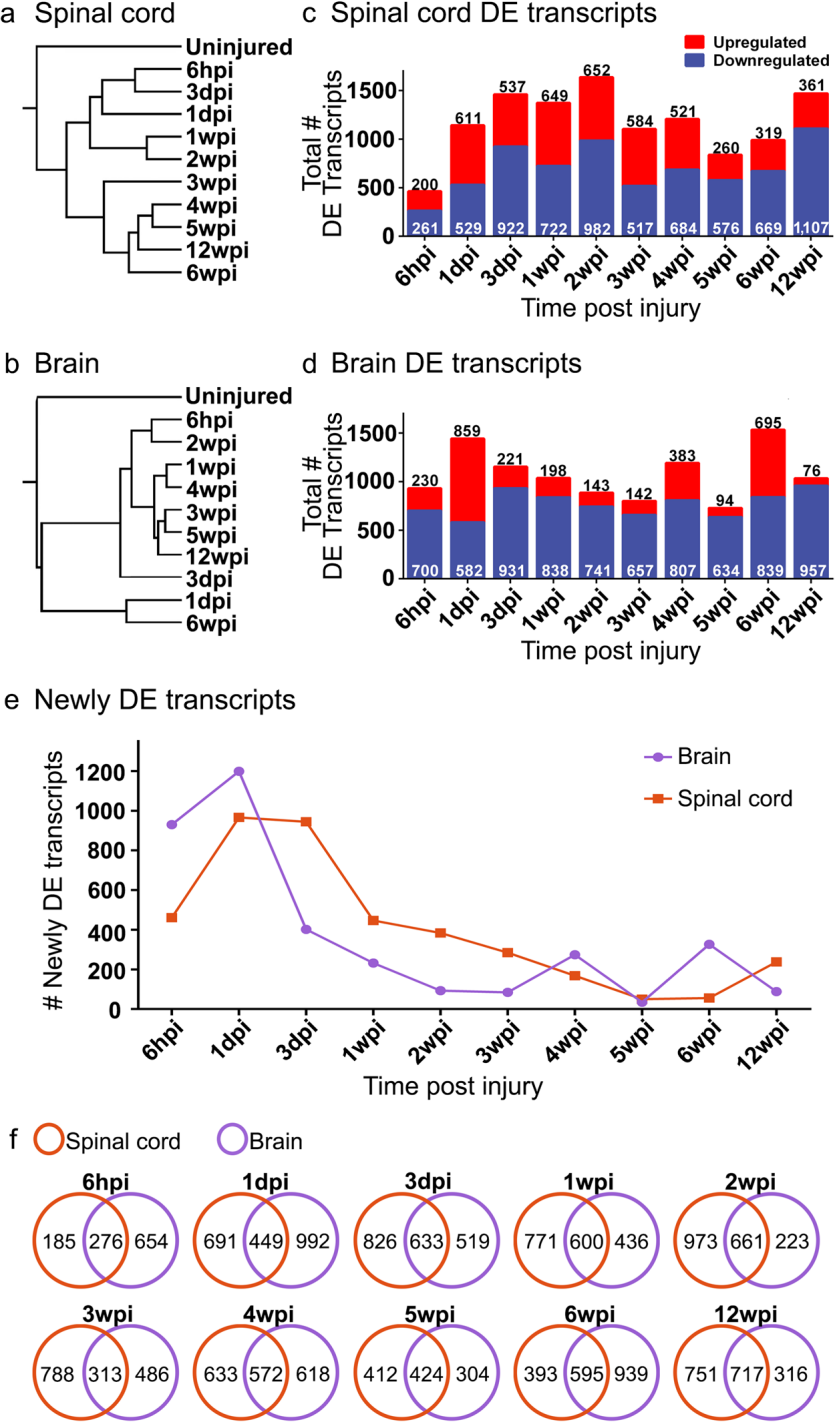
Differential expression of transcripts after spinal cord injury. (**a**,**b**) Dendrograms depict 2-way hierarchical clustering (Ward method) to reveal relationships between transcriptional profiles at experimental time points in spinal cord (**a**) and brain (**b**). The number of transcripts that were differentially expressed is shown for spinal cord (**c**) and brain (**d**). (**e**) Line graphs show number of transcripts that are newly differentially expressed at each time point following injury. (**f**) Venn diagrams show the number of transcripts among differentially expressed transcripts that are shared or unique to spinal cord or brain at each time point. Transcripts corresponding to each panel in F are found in Supplementary Table S3.

To determine broad patterns of transcript expression following SCI, we used 2-way hierarchical clustering of expression profiles of spinal cord and brain. In both tissues, the gene expression profiles of the uninjured condition clustered separately from all time points obtained post injury (Fig. 2a,b). In the spinal cord, the clustering of expression profiles reflected progression through the time course of functional recovery, such that acute time points (6hpi to 3dpi) clustered separately from later time points (3 to 12wpi) (Fig. 2a). In contrast, hierarchical clustering of gene expression profiles in brain did not similarly reflect the progression through the time course of functional recovery (Fig. 2b). Notably, expression profiles at 1dpi and 6wpi formed a separate, distinct cluster. Transcriptional profiles at 12wpi in both spinal cord and brain clustered with profiles from other post-injury time points, indicating that altered gene expression occurred even in late stages of functional recovery and not a return to the uninjured state (Fig. 2a,b).

Next, we identified transcripts that were differentially expressed after SCI, relative to the uninjured condition, using EBSeq^36^. These analyses revealed robust and dynamic changes in gene expression in spinal cord and brain throughout the experimental time course (Fig. 2c,d). Notably, a distinct wave of newly differentially expressed transcripts was observed during the first week after SCI, corresponding to the onset of wound healing and tissue morphogenesis, but newly differentially expressed transcripts were detected throughout the course of functional recovery (Fig. 2e). This continued even into late stages of functional recovery (12wpi), at which point 238 and 88 newly differentially expressed transcripts were observed in spinal cord and brain, respectively (Fig. 2e; see Supplementary Table S1). Thus, dynamic changes in gene expression persist throughout the time course of recovery after SCI, even at late stages of behavioral recovery. We observed both shared and tissue-specific responses in expression at all post-injury time points (Fig. 2f; see Supplementary Table S3). This suggests that there are both global transcriptional responses to SCI and more locally tuned responses that reflect intrinsic differences in cell type and varying distances of the spinal cord and brain tissue from the lesion site.

**Figure 3:**
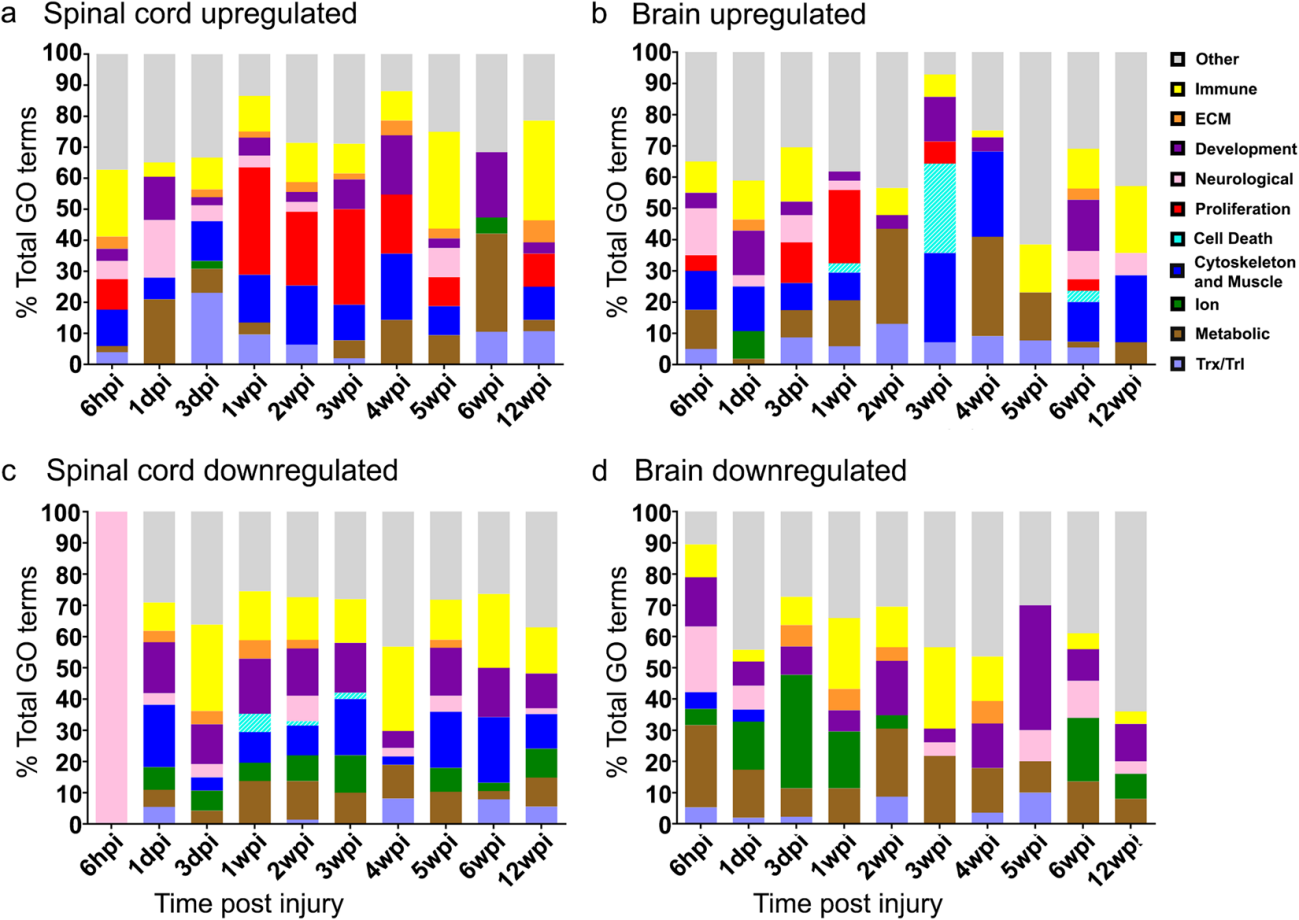
Functions of differentially expressed genes. Stacked bar graphs show the percentages (Y-axis) of significantly enriched categories of GO Biological Process (BP) terms encompassing differentially expressed transcripts that are upregulated (**a**,**b**) or down-regulated (**c**,**d**) in the spinal cord or brain at each time point (p ≤ 0.05). The X-axis indicates the experimental time point. Individual GO terms were manually bundled into larger descriptive groups (see Supplementary Table S4), indicated in the legend.

We leveraged functional data from human and mouse to identify likely functions of differentially expressed transcripts using Enrichr, an open bioinformatics platform that utilizes mammalian data^37^. Analysis of statistically enriched (p < 0.05) Gene Ontology (GO) categories encompassing differentially expressed transcripts indicated complex transcriptional responses in spinal cord and brain at most time points after SCI (Fig. 3a–d; see Supplementary Table S4). Broad functional categories of differentially expressed transcripts were related to immune function, extracellular matrix (ECM) remodeling/deposition, development, neuronal function, proliferation, cell death, cytoskeleton, ion channels, metabolism and transcription/translation (Fig. 3a–d). Previous histological studies of lampreys after SCI showed that cell proliferation occurs in the brain and even more so in the spinal cord, and that neuronal death increases in brain by 3wpi^28–31^. In agreement with these data, here we found that upregulated transcripts related to cell proliferation were more abundant in spinal cord than brain, while those related to cell death peaked in brain at 3wpi (Fig. 3a,b). Evaluation of differentially expressed transcripts using the Kyoto Encyclopedia of Genes and Genomes (KEGG, www.kegg.jp/kegg/kegg1.html) database indicated similar themes related to immune, ECM, development, neurological, and cytoskeletal processes (see Supplementary Table S5). Taken together, these analyses revealed transcriptional changes in broad functional gene categories after SCI in lampreys that have also been observed in other highly regenerative vertebrate species, such as zebrafish and axolotls, as well as in regenerating mammalian PNS^3,38–40^.

**Figure 4:**
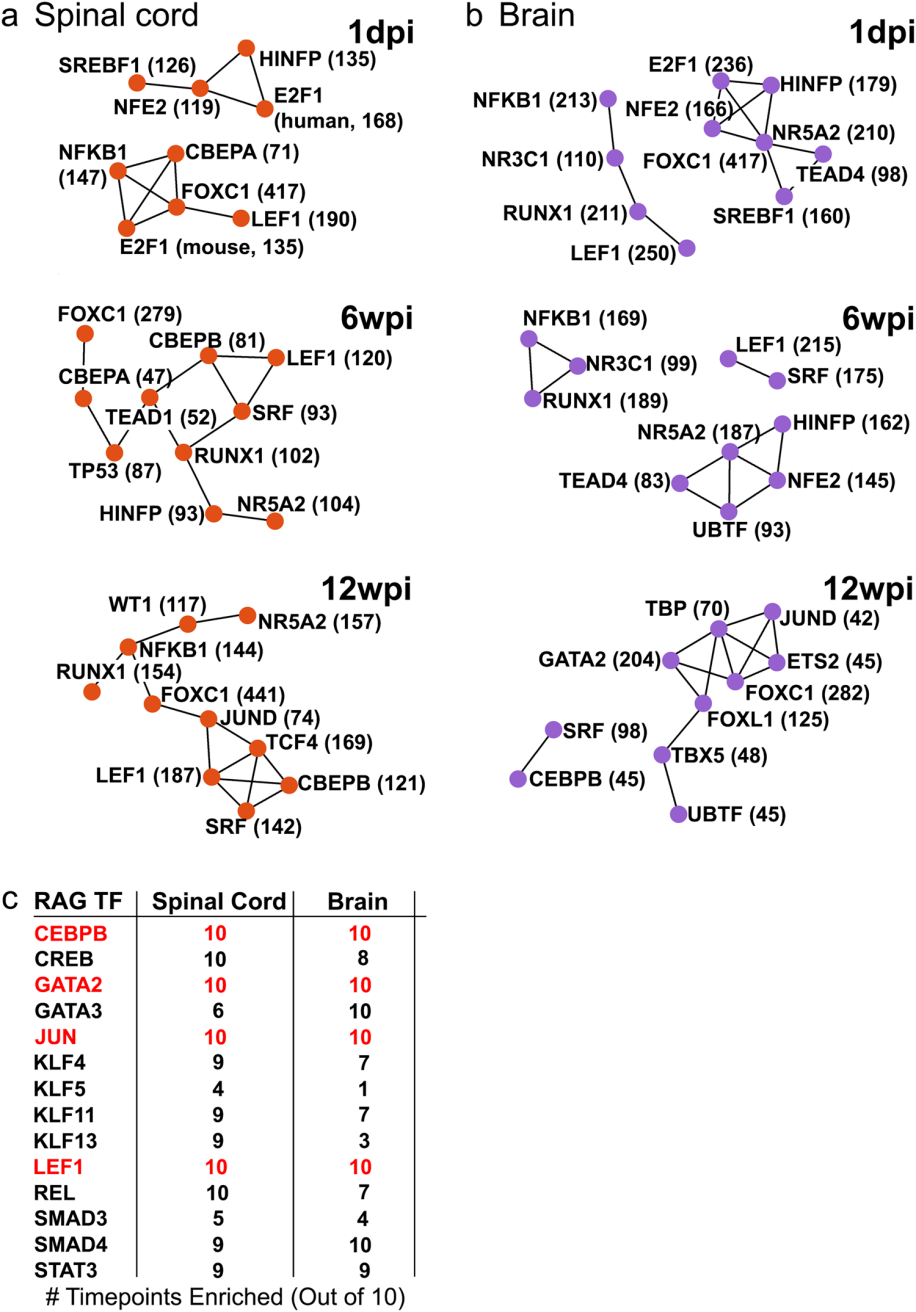
Transcription factors that are predicted to regulate differentially expressed genes. TRANSFAC/JASPAR was used to infer the transcription factors (TFs) that regulate differentially expressed genes at each time point. TF networks generated by TRANSFAC/JASPAR within Enrichr are shown for spinal cord (**a**) and brain (**b**) at 1dpi, 6wpi, and 12wpi. Values shown next to TFs indicate the number of targets that are differentially expressed at that time point. Only the most significant (Fisher’s exact test) TFs are shown for each network. (**c**) Regeneration associated genes (RAGs) predicted by TRANSFAC/JASPAR to regulate differentially expressed genes. Numbers indicate number of time points when that TF is significantly enriched (P ≤ 0.01, Fisher’s exact test). Red indicates the RAGs whose targets were differentially expressed in both tissues at all 10 post-injury time points, which include targets of the Wnt pathway.

To identify transcription factor (TF) networks that could potentially coordinate molecular responses to SCI in lampreys, we used TRANSFAC/JASPAR to assign the differentially expressed transcripts to TFs known to regulate expression of mammalian homologues. TF networks that regulate the most highly expressed transcripts in uninjured spinal cord and brain are shown in Supplementary Fig. S1. We then analyzed TF networks regulating differentially expressed transcripts after SCI at 1dpi, 6wpi, and 12wpi, corresponding to acute, intermediate, and late phases of functional recovery (Fig. 4). Enriched TF networks differed between tissues at each time point and changed dramatically over time within each tissue (Fig. 4a,b). Notable TFs whose targets were enriched after SCI included: FOXC1, which has been implicated in embryonic development; NFKB1, a master regulator of the immune system and growth factors, and LEF1, a member of the canonical Wnt pathway, which has been implicated in tissue regeneration in several species (Fig. 4a,b)^41,42^. In addition, 17 TFs with targets enriched amongst differentially expressed genes were previously identified in the mammalian PNS as regeneration-associated genes (RAGs) (Supplemental Table S7), 12 of which are shown in Fig. 4c. Several of these RAGs belong to canonical and non-canonical Wnt signaling pathways (e.g. JUN, LEF1, SMAD) (Fig. 4c)^40–42^. Interestingly, there were 4 RAGs in the TF networks regulating differentially expressed transcripts in both tissues at all ten post-injury time points: CEBPB, GATA2, JUN, and LEF1 (Fig. 4c-red). Other RAGs with targets enriched amongst differentially expressed transcripts included KLF, SMAD, and STAT family members, which also intersect with Wnt signaling pathways^41–43^. These data indicate that the lamprey CNS utilizes highly conserved TF networks and signaling pathways that support neural repair and regeneration in the mammalian PNS and in other non-mammalian vertebrates that regenerate after SCI and suggest a role for Wnt signaling^40,44–48^.

**Figure 5:**
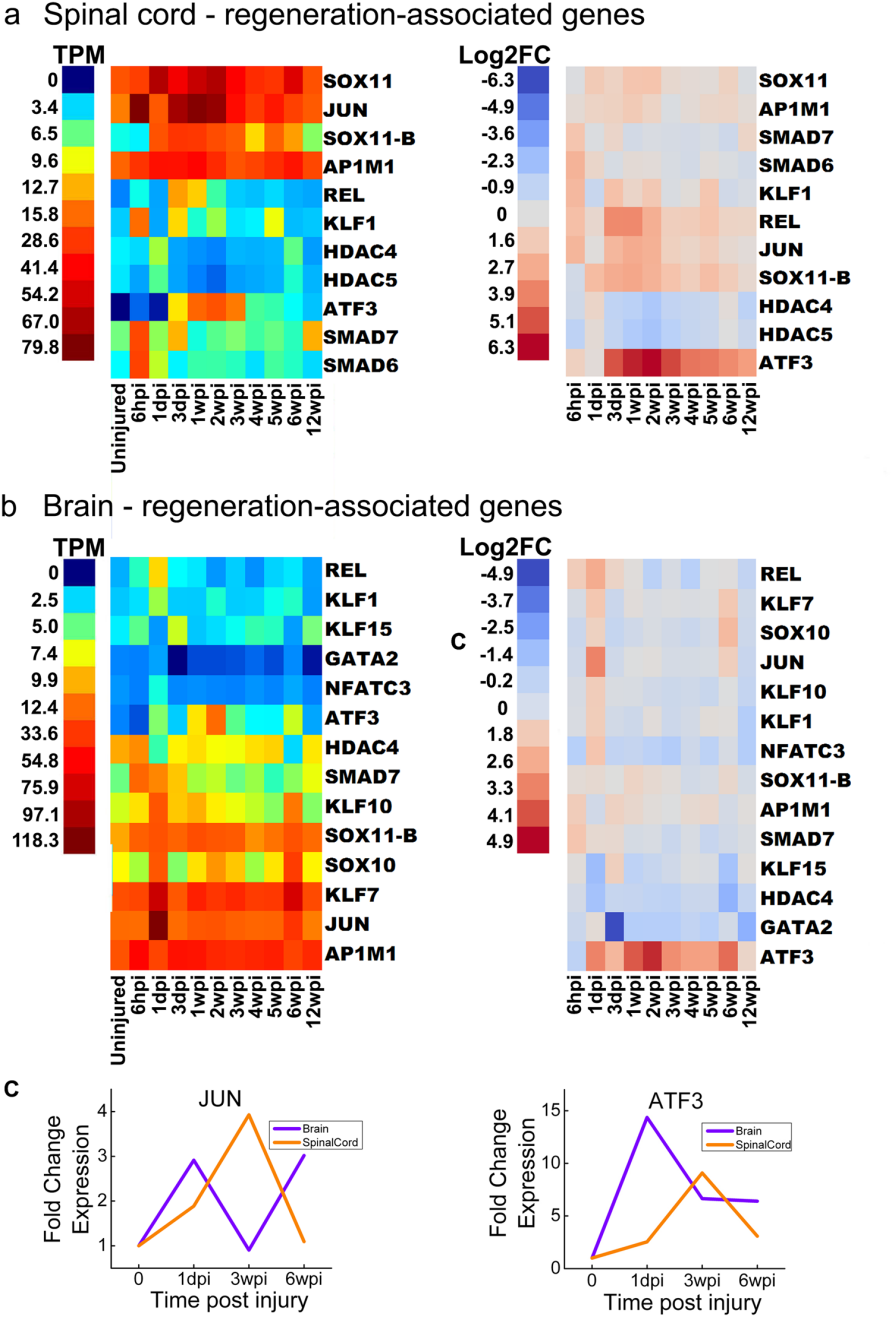
Temporal expression patterns of regeneration associated genes (RAGs). Heat maps generated from RNA-Seq data showing expression of genes that were differentially expressed at least once during the experimental time course for spinal cord (**a**) and brain (**b**). Left panels show expression data and right panels show log_2_-fold changes (FC) relative to the uninjured state. TPM = transcripts per million reads. Lamprey gene IDs and corresponding gene symbols are found in Supplementary Table S2. (**c**) qPCR data showing expression changes for JUN and ATF at 1dpi, 3wpi, and 6wpi, compared to uninjured controls (time 0).

We next examined SCI-induced expression changes in the RAGs themselves (Fig. 5)^40,44,45^. In the uninjured spinal cord and brain, individual RAGs were expressed at varying levels (Fig. 5a,b, left). SCI induced significant upregulation of most RAGs at one or multiple time points in both spinal cord and brain, beginning as early as 6hpi (Fig. 5a,b, right). As expected, differentially expressed RAGs included many of the specific TFs that were predicted independently in the TRANSFAC analysis, including RAGs that intersect with Wnt signaling: JUN, KLF, REL, and SMAD family members (Fig. 5a,b). Interestingly, ATF3, a member of the CREB TF family that promotes axon regeneration in mammalian PNS, as well as central axon branches in dorsal root ganglion neurons, was the most robustly induced RAG in both spinal cord and brain after SCI (Fig. 5a,b)^40,49,50^. In addition, homologs of JUN, SOX11, SMAD and REL, which along with ATF3, are positioned centrally within the core TF networks associated with mammalian PNS injury responses, were highly upregulated in spinal cord (Fig. 5)^40^. In the brain, several RAGs were significantly upregulated specifically at 1dpi and 6wpi (Fig. 5b) reflecting broader patterns identified through hierarchical clustering (Fig. 2a). The temporal expression profiles for JUN and ATF3 were further validated using qPCR, including the upregulation of JUN at 1dpi and 6wpi in brain (Fig. 5c). Together, these data show activation of highly conserved molecular pathways in the lamprey after SCI that also accompany regeneration in the mammalian PNS.

We also examined gene expression profiles for other gene families commonly studied in the context of PNS or CNS regeneration, including axon growth and guidance (Supplementary Fig. S2), extracellular matrix (Supplementary Fig. S3), cell proliferation/death (Supplementary Fig. S4), ion channels (Supplementary Fig. S5), and immune function (Supplementary Fig. S6). Broadly speaking, many axonal growth-promoting transcripts were upregulated after SCI (*e.g*. SNAP25, activin receptor ACVR1), while growth-restricting transcripts were downregulated (*e.g*. RHOB, SLIT2/3, ROBO2/3) (Supplementary Fig. S2). We also observed an upregulation of neurofilaments (NEFH/L) and synapsin (SYN2/3) gene family members in the brain, as well as differential expression of semaphorins and their receptors (SEMA3/4/5, PLXN) in both tissues, which is in agreement with previously published lamprey SCI studies that used *in situ* hybridization to detect expression changes (Supplementary Fig. S2)^32,51–53^. Transcripts associated with ECM deposition and remodeling were generally upregulated [*e.g*. collagens (COL), laminins (LAM), ADAMs, TIMPs] (Supplementary Fig. S3). Reflecting ongoing tissue repair, many cell proliferation related transcripts were highly upregulated after SCI in both spinal cord and brain (Supplementary Fig. S4)^16,18^. Interestingly, synuclein (SNCA) (Supplementary Fig. S4), a gene whose overexpression or mutation is linked to neurodegeneration in Parkinson’s disease, is upregulated in the spinal cord after SCI^54,55^. This increased expression may contribute to the accumulation of synuclein protein that occurs in a subset of lamprey neurons after SCI and leads to neurodegeneration, which we previously reported^31,56^. Several ion channel transcripts were also differentially expressed after SCI, likely reflecting substantial changes in neuronal excitability during regeneration and functional recovery (Supplementary Fig. S5). In mammals, the immune system has both beneficial and detrimental effects after SCI^57,58^. In lamprey, robust immune system responses were observed, with cytokines (e.g. IL8) and chemokines (e.g. CXCR4) and prostaglandins, upregulated acutely after SCI (Supplementary Fig. S6). However, this does not inhibit the robust regeneration and functional recovery that is normally achieved in these animals (see Fig. 1). Notably, upregulation of prostaglandins after traumatic brain injury in zebrafish has been previously shown to enhance tissue repair and recovery^59^. Though this is far from conclusive, these data are consistent with a permissive or positive role for immune responses in recovery from SCI in lampreys.

### Wnt Signaling Pathways are differentially expressed after SCI and Necessary for Functional Recovery

Wnt pathways are involved in a variety of biological processes that may be relevant to SCI, including body plan patterning, contact-dependent signaling, cell proliferation, tissue development and regeneration, stem cell self renewal, and axon guidance^41–43,60–62^. There are 19 mammalian Wnt genes, many of which are conserved across species (including invertebrates) and interact promiscuously with their multimeric receptors involving Frizzled and LRRP proteins. Porcupine (PORCN) is an O-acyltransferase required for Wnt palmitoylation, maturation, and subsequent secretion^63,64^. Due to the important role of Wnts in various disease settings, including cancer, several small molecules have been identified that target PORCN or other Wnt pathway members^60,63,64^. The role of Wnt signaling after SCI in mammals is complex and still controversial given the observation of highly variable results among different rat and mouse models^42,43,65–67^. However, Wnts appear to be pro-regenerative after SCI in zebrafish and after tail injury in salamander^41,48,68,69^. In fact, Voss and colleagues recently demonstrated that incubation in C59, a drug that inhibits *PORCN*, blocked salamander tail regeneration^63,69^.

**Figure 6:**
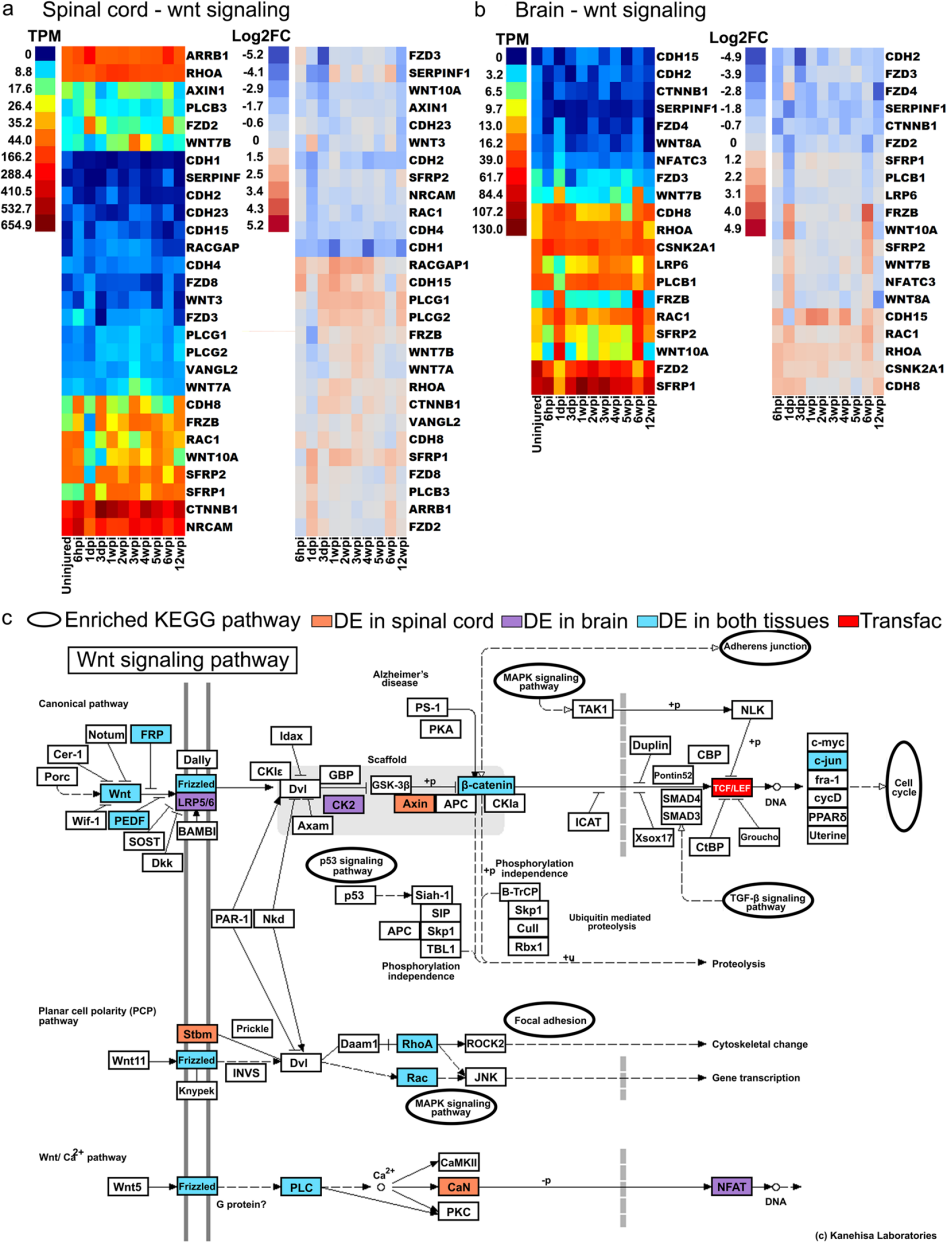
Differential expression of Wnt signaling pathway genes after spinal cord injury. (**a**,**b**) Canonical and non-canonical Wnt signaling pathways are shown. Genes that were differentially expressed at least once during the experimental time course are indicated in red (up) or blue (down) regulated. Heat maps showing expression of genes that were differentially expressed at least once during the experimental time course for spinal cord and brain. Left panels show raw expression data and right panels show log_2_-fold changes (FC) relative to the uninjured state. TPM = transcripts per million reads. Lamprey gene IDs and corresponding gene symbols are found in Supplementary Table S2. (**c**) KEGG analysis of the Wnt signaling pathways showing transcripts identified as differentially expressed in spinal cord (orange), brain (purple) or both (blue). KEGG pathway map 04310 is adapted here from http://www.kegg.jp/kegg/kegg1.html. The KEGG database has been described previously^93–95^.

**Figure 7:**
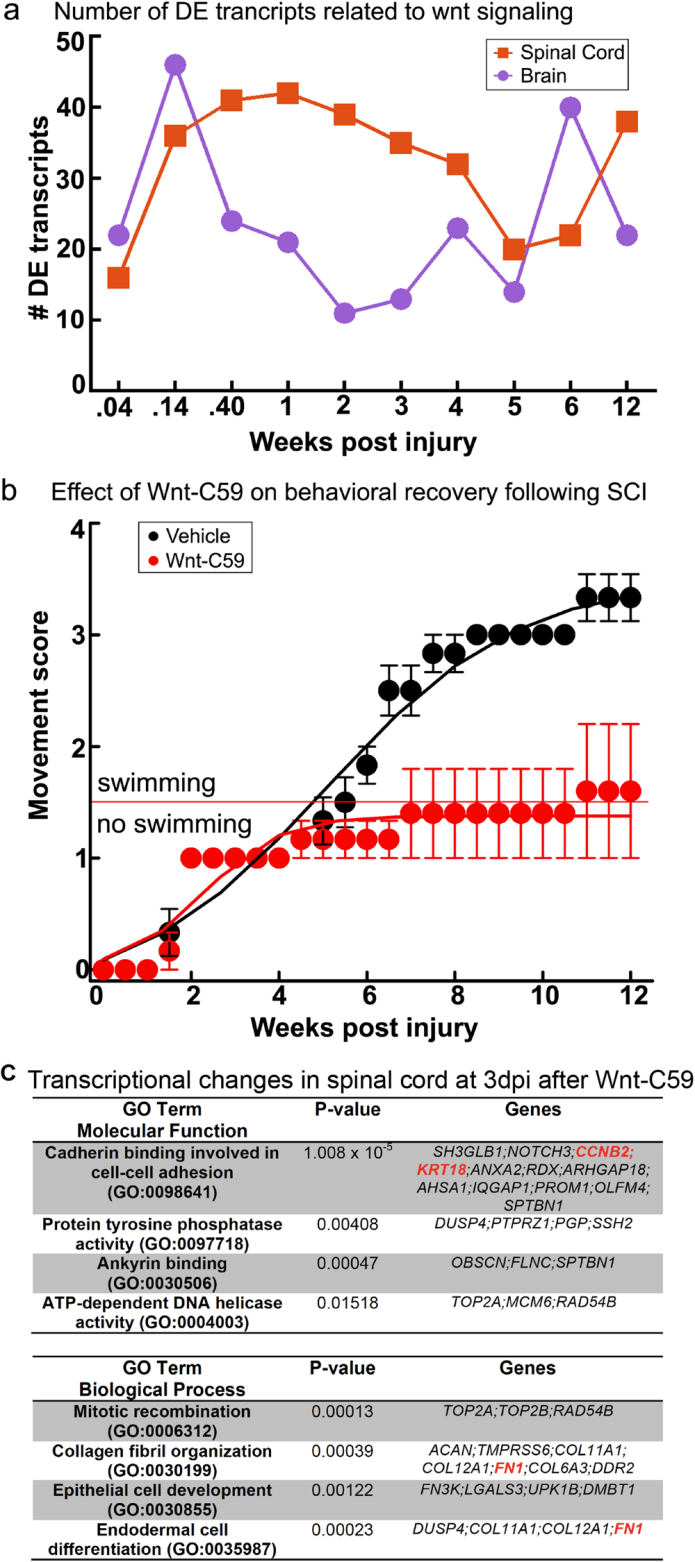
Blocking Wnt signaling inhibits functional recovery after complete spinal cord transection in lampreys. (**a**) Number of differentially expressed transcripts related to Wnt signaling over time. Lamprey gene IDs and corresponding gene symbols are shown in Supplementary Table S6. (**b**) Functional recovery (swimming) is inhibited in animals that were treated with Wnt-C59 (red), as compared to animals that were treated with vehicle (black) at the time and site of transection. Functional recovery was scored as in Fig. 1. Data points represent mean ± SEM (n = 5–6 animals per time point). (**c**) Transcriptional responses in spinal cord at 3dpi after C59 treatment. Gene ontology (GO) analysis is consistent with changes in cell adhesion, differentiation and proliferation.  Gene symbols in red indicate direct targets of the Wnt pathway. See also Supplementary Table S6.

As discussed above, some of the RAGs (e.g. JUN) and other differentially expressed transcripts belong to or intersect with Wnt signaling pathways, both canonical (beta-catenin dependent) or non-canonical (beta-catenin independent) pathways. We identified a total of 121 and 101 differentially expressed transcripts related to Wnt signaling in spinal cord and brain, respectively (Supplementary Table S6). Amongst these were transcripts encoding for a number of Wnt and Frizzled family members, as well as proteins involved in intracellular Wnt signaling (Fig. 6a,b). KEGG analysis revealed how extensively the Wnt signaling pathways were represented amongst the differentially expressed transcripts in spinal cord and brain, as well as TCF/LEF1 that was identified by TRANSFAC/JASPR in the transcription factor networks (Fig. 6c). The number of Wnt related transcripts peaked acutely within the first week post-injury in both spinal cord and brain with additional increases at later time points (Fig. 7a). In light of these data, we decided to directly assess whether Wnt signaling has any effect on functional recovery after SCI in lampreys. We applied a single dose of the PORCN inhibitor C59 (10 μM) or vehicle to the spinal cord at the time and site of transection. Since *porcupine* is required for Wnt palmitoylation, secretion, and therefore activity, C59 potently inhibits Wnt signaling^63^. We then scored swimming behavior for 12 weeks, as in Fig. 1c. At 12wpi, when lampreys typically achieve full recovery, the animals that received C59 achieved an average movement score of 1.6 ± 0.6 and thus remained non-swimming or exhibited only brief uncoordinated swimming, while vehicle-treated animals achieved an average score of 3.33 ± 0.2 and swam in a coordinated sinusoidal wave (Fig. 7b).

To determine the molecular basis of these functional effects, and to confirm the efficacy of C59 in lampreys, we performed RNA-Seq on C59- and vehicle-treated lampreys at 3dpi, a period of rapid and dynamic change in the transcription of Wnt-responsive genes (Fig. 7a). Gene ontology analysis revealed significant expression changes in spinal cord transcripts that were related to cadherin and ankyrin binding; DNA replication and mitotic recombination; collagen and fibrin organization; and cell development and differentiation, all of which are affected by Wnt signaling (Fig. 7c)^70,71^. Individual transcripts that were differentially expressed in spinal cord after C59 treatment included direct targets of the canonical Wnt signaling pathway, such as cadherin (CDH2), claudin (Cldn19), cyclin (CCNB2), fibronectin (FN1), and keratins (KRT6a/8/18) (Fig. 7c; Supplementary Table S6). In brain, the differentially expressed transcripts included additional targets of Wnt signaling, such as axin (Axin1), Jagged (JAG2), JUN, keratins (KRT7/10), matrix metalloproteinase (MMP9), and T-box transcription factors (Tbr1; TBX20) (Supplementary Table S6). These data indicate that Wnt signaling plays a positive and crucial role early on during the process of functional recovery after SCI in lampreys, likely through effects on cell adhesion and proliferation.

## Discussion

Large-scale gene expression analyses are rapidly advancing our understanding of molecular responses in vertebrates to nervous system injury^38,40,72^. For example, a microarray study of mammalian cortical neurons revealed developmentally regulated genes promoting axon outgrowth and an RNA-Seq study showed that in comparison to mammalian CNS neurons, PNS neurons had greater expression of pro-regenerative genes (i.e. growth factors)^44,73^. Molecular responses to SCI in zebrafish, which recover after SCI, resembled injury responses in the mammalian PNS^5,38,40^. Similarly, in frogs and salamanders, SCI induced rapid molecular responses that support neural regeneration^39,74^. Despite these advances in knowledge, there are a number of fundamental questions that still need to be addressed. First, what are the most conserved molecular mechanisms that support spinal cord regeneration and functional recovery^2^? This requires unbiased molecular profiling of CNS regeneration in a basal vertebrate that recovers after SCI, such as the lamprey. Second, how do the molecular mechanisms that support functional CNS regeneration change over time? This requires a denser temporal sampling after SCI than has been reported previously, spanning the entire time course of recovery. Third, how extensive are the molecular responses to SCI in the brain, which contains the cell bodies of injured neurons with descending axons? This requires parallel analyses of supraspinal molecular responses after SCI, which has been greatly understudied. While recent studies in mammals have yielded exciting potential strategies such as manipulating microtubule dynamics, enhancing successful regeneration and functional recovery after SCI in mammals remains challenging, in part because our understanding of the conserved molecular pathways that promote these processes successfully in other vertebrate species and biological settings is incomplete^10^.

Here, we addressed these three questions by performing the first RNA-Seq analysis on spinal cord and brain during regeneration after SCI in the sea lamprey, a vertebrate that provides a critical perspective on the deep ancestry of all living vertebrates. The lamprey has long been appreciated to have the amazing capacity to regenerate and recover function after complete spinal cord transection. However, a relative paucity of molecular tools in the lamprey has largely limited studies of the molecular mechanisms underlying these abilities to a candidate gene approach^33,51,52,75–77^. The increasing ease of performing RNA-Seq, which uses direct sequencing without prior sequence information, is aiding a greater understanding of molecular responses in species with historically limited genomic resources such as lamprey^12,78,79^.

Results from this study reveal several key findings. First, these data reinforce lessons from the recent publication of the lamprey genome, which is that the lamprey CNS expresses homologs of a large number of mammalian CNS genes, indicating a high degree of molecular conservation across vertebrates^12^. Second, SCI in lamprey induces expression of many transcripts associated with regeneration in the mammalian PNS, illustrating the power of this organism as a model for identifying and studying highly conserved, fundamental, pro-regenerative molecular pathways^3,38–40,44^. Third, SCI induces rapid, robust, and long-lasting changes in gene expression in the brain, implicating supraspinal responses as a major component of anatomical and functional recovery. Fourth, late stages of functional recovery occur in a novel molecular context and are not a simple restoration to the uninjured transcriptional program. Fifth, Wnt signaling is necessary for functional recovery in the lamprey after SCI, justifying future studies aimed at elucidating the required aspects of the Wnt pathway.

One of the most surprising findings from this study is the robust and complex transcriptional responses occurring in the lamprey brain after SCI. Previous studies in lamprey revealed SCI-induced changes in expression levels of genes related to axon growth and guidance within the RS neuron somata located in the brain and in the tips of regenerating axons^33,51,52,76,77,80^. Here, we confirmed and extended these observations to demonstrate global supraspinal changes in gene expression throughout the course of functional recovery. As data increasingly indicate that it is possible to promote functional recovery in persons in the chronic phase of SCI, understanding supraspinal molecular responses that are consistent with neuroplasticity and regeneration, such as those described here, will be of increasing importance^81,82^.

Multiple independent bioinformatics analyses revealed that RAGs, many of which are members of the canonical and non-canonical Wnt signaling pathways, are differentially expressed after SCI in lamprey. ATF3 was the most highly induced RAG in both tissues (Fig. 5). In mammals, ATF3 enhances neurite outgrowth, partly by promoting effects of JUN^49,83^. Microarray analyses also revealed that ATF3 is upregulated after SCI in zebrafish^38^. Both ATF3 and JUN were identified as synergistic hubs in TF networks that promote regeneration of mammalian dorsal root ganglion neurons, peripheral neurons with high regenerative potential^40^. In mammals, Wnts plays a role in axon growth during development. However, the role of Wnts after SCI is still unclear because different injury models and use of multiple rodent species have resulted in conflicting data suggestive of both positive and negative roles^41–43,65–67^. As mentioned earlier, Wnts promote regeneration and recovery from injury in several non-mammalian vertebrates, including zebrafish, salamanders^48,68,69^ and now lampreys. Interestingly, Wnts were also recently shown to be differentially expressed during head regeneration in a hemichordate, suggesting that their functional roles are deeply evolutionarily conserved^84^. Future experiments are needed to determine the mechanism by which Wnt signaling promotes functional recovery, including an analysis of the affected cellular and molecular pathways.

Despite these important new insights, we acknowledge several limitations to this study. The current lamprey reference transcriptome is incomplete, and future iterations will incorporate germline-specific regions and more diverse transcriptomic datasets^12^. Also, the tools for functional genetic analyses in lamprey are not as well developed as in other SCI models. However, gene overexpression, morpholino and CRISPR/Cas9 knockdowns, as well as pharmacological agents, have already been used successfully to manipulate gene expression in lampreys, providing multiple avenues for additional functional analyses^53,56,85–89^. Furthermore, although we present here a complex molecular profile of transcripts expressed in the entire brain and spinal cord, which contains a wide array of cell types, future analyses will be greatly facilitated by determining the molecular programs that are specific to individual cell types or brain regions. Lastly, we used an inhibitor that targets secretion of all Wnts, so the precise components of this pathway needed to support regeneration after SCI requires further inquiry. Despite these considerations, this study provides broad mechanistic insights into conserved molecular pathways, including Wnt signaling, that promote anatomical and functional recovery after SCI in vertebrates.

## Methods

Experiments were performed on late larval sea lampreys (*Petromyzon marinus*, 10–13 cm; ~5–7 years old), in accordance with institutional IACUC regulations and included in experimental protocols approved by The Marine Biological Laboratory and The Feinstein Institute for Medical Research. Spinal cord transections, behavioral recovery scoring, and tissue histochemistry were performed as described previously^18,27^. To block Wnt signaling, Wnt-C59, (Selleckchem; Houston, TX) was added (10μM in 0.1% DMSO/PBS) at the time and site of spinal injury via a small piece of Gelfoam (Pfizer, NY, NY). Animals undergoing spinal cord injury and treated with vehicle (0.1% DMSO/PBS) were used as controls. These manipulations slowed the trajectory of functional recovery (Fig. 7b-vehicle), compared to recovery without any manipulations (Fig. 1c), which is due to insertion of the Gelfoam.

### RNA-Seq library preparation and sequencing

Brains (whole brains without olfactory lobes) and spinal cords (1 cm surrounding the lesion), were harvested from uninjured lampreys, and at 10 time points from 6 hours to 12 weeks post injury (Fig. 1a). Tissue was stored in RNALater and then homogenized, pooled by time point (n = 6 animals per time point), and total RNA (RIN > 8.4) extracted using Trizol. RNA-Seq libraries were created using TruSeq RNA Sample Prep Kit v2 (Illumina Inc., San Diego, CA), + Ribo-Zero Gold (Epicentre). RNA-Seq libraries (~250 bp) were sequenced using 100 bp paired end (pe) reads on the Illumina HiSeq Platform at the Genomics Core, Icahn School of Medicine at Mount Sinai (New York, NY).

### Statistical analysis of RNA-Seq data

The average number of RNA-Seq reads per timepoint was 135,742,182 in spinal cord and 139,024,579 in brain. Total RNA-Seq reads for all time points was 1,493,164,006 for the spinal cord and 1,529,270,374 for the brain. RNA-Seq reads were mapped against gene models from the publicly available lamprey genome (Ensembl; Pmarinus_7.0)^12^. Gene expression levels were estimated for all lamprey gene models separately for all time points, and tissues and were quantified from each time point using RNA-Seq by Expectation-Maximization (RSEM)^90^. The expression values (TPM) for all 24,272 gene models are provided in Table S1. Within the lamprey genome, more than one gene may be the presumptive homolog of a specific human or mouse gene and may represent split gene models or gene duplication events (recent and ancient)^12,91^. For preparation of figures, a single homolog was selected that maximized transcript length, expression (transcripts per million, TPM), and amino acid sequence identity with the corresponding mammalian homolog. BLAST results for a sample of transcripts are shown in Table S2. For clarity, data presented in the figures focus only on the annotated transcripts, unless otherwise specified. Following estimation of expression values, EBSeq was used to identify differentially expressed genes throughout the experimental time course^36,92^. Fold change estimates and corresponding Bayesian statistics are presented in Table S1. Genes were considered of interest for further analysis if they were determined to be differentially expressed by EBSeq with a posterior probability of differential expression (PPDE) of ≥0.95. JMP Statistical software (SAS) was used to create heat maps and to perform hierarchical clustering. To illustrate relative changes in transcript expression (Figs 5 and 6, Supplementary Figures S2–S6), fold change (FC) relative to uninjured values shown is the −Log_2_(PostFC) transformation of values generated by EBSeq. Deep sequencing and processed data sets were deposited in Gene Expression Omnibus.

### Enrichment analysis of RNA-Seq data

Genes were analyzed using the program Enrichr^37^. Enrichr uses official human and mouse gene symbols for its input data, and symbols used in our analyses were assigned on the basis of the published set of lamprey gene annotations^12^. GO terms that were concatenated for simplicity into larger related categories or categorized as “Other” in Fig. 3 are available in Table S4.

## Electronic supplementary material

Supplemental Information

Supplementary Table S1

Supplemental Table S2

Supplemental Table S3

Supplemental Table S4

Supplemental Table S5

Supplemental Table S6

Supplemental Table S7

Supplementary Video
