# Supplementary material for: Highly conserved molecular pathways, including Wnt signaling, promote functional recovery from spinal cord injury in lampreys
Source: Sci Rep. 2018 Jan 15;8:742. doi: 10.1038/s41598-017-18757-1 (PMC5768751; doi:10.1038/s41598-017-18757-1)
Supplement: Supplementary file 1 — Supplemental Information [file 41598_2017_18757_MOESM1_ESM.pdf]

# Highly conserved molecular pathways, including Wnt signaling, promote functional recovery from spinal cord injury in lampreys

**Paige E. Herman[1,+], Angelos Papatheodorou[1,+], Stephanie A. Bryant[2], Courtney K.M. Waterbury[2], Joseph R. Herdy[2], Anthony A. Arcese[1], Joseph D. Buxbaum[3], Jeramiah J. Smith[2], Jennifer R. Morgan[4,*], Ona Bloom[1,*]**

1. The Feinstein Institute for Medical Research; Center for Autoimmune and Musculoskeletal Disease, Manhasset, NY 11030, USA

2. University of Kentucky; Department of Biology, Lexington, KY, 40506, USA

3. Icahn School of Medicine at Mount Sinai; Department of Psychiatry; New York, NY 10029, USA

4. Marine Biological Laboratory, The Eugene Bell Center for Regenerative Biology and Tissue Engineering; Woods Hole, MA 02543, USA

+Equally contributing authors

*Equally contributing corresponding authors

## SUPPLEMENTARY INFORMATION

### Supplementary Figure Legends

**Supplementary Figure S1.** Transcription Factors that Regulate Highly Expressed Transcripts in Uninjured Animals. TRANSFAC/JASPAR was used to determine the TFs that regulate transcripts expressed at 10X the mean (TPM) in uninjured animals. Networks show the most significant transcription factors ($p<0.01$, Fisher's exact test).

**Supplementary Figure S2.** Temporal expression patterns of differentially expressed transcripts that are related to axon growth and guidance. Heat maps showing expression of genes that were differentially expressed at least once during the experimental time course for spinal cord (A) and brain (B). Left panels show expression data and right panels show $\log_2$-fold changes (FC) relative to the uninjured state. TPM = transcripts per million reads. Lamprey gene IDs and corresponding gene symbols are found in Table S2.

**Supplementary Figure S3.** Temporal expression patterns of differentially expressed transcripts that are extracellular matrix components. Heat maps showing expression of genes that were differentially expressed at least once during the experimental time course for spinal cord (A) and brain (B). Left panels show expression data and right panels show $\log_2$-fold changes (FC) relative to the uninjured state. TPM = transcripts per million reads. Lamprey gene IDs and corresponding gene symbols are found in Table S2.

**Supplementary Figure S4.** Temporal expression patterns of differentially expressed transcripts that are related to cell proliferation and cell death. Heat maps showing expression of genes that were differentially expressed at least once during the experimental time course for spinal cord (A, C) and brain (B, D). Left panels show expression data and right panels show $\log_2$-fold changes (FC) relative to the uninjured state. TPM = transcripts per million reads. Lamprey gene IDs and corresponding gene symbols are found in Table S2. (E) qPCR data demonstrating expression of BAG2 in brain (purple) and spinal cord (orange).

**Supplementary Figure S5.** Temporal expression patterns of ion channels that are differentially expressed during spinal cord regeneration. Heat maps showing expression of genes that were differentially expressed at least once during the experimental time course for spinal cord (A) and brain (B). Left panels show expression data and right panels show $\log_2$-fold changes (FC) relative to the uninjured state. TPM = transcripts per million reads. Lamprey gene IDs and corresponding gene symbols are found in Table S2.

**Supplementary Figure S6.** Temporal expression patterns of genes related to immune response. Heat maps showing expression of genes that were differentially expressed at least once during the experimental time course for spinal cord (A) and brain (B). Left panels show expression data and right panels show $\log_2$-fold changes (FC) relative to the uninjured state. TPM = transcripts per million reads. Lamprey gene IDs and corresponding gene symbols are found in Table S2. (E) qPCR data demonstrating expression of CASP3 in brain (purple) and spinal cord (orange).

## Supplementary Tables

**Supplementary Table S1.** Expression Data and Analysis.

**Supplementary Table S2.** Lamprey Gene ID lookup table and BLAST results for data displayed in heat maps.

**Supplementary Table S3.** Differentially expressed transcripts are unique or shared in spinal cord and brain.

**Supplementary Table S4.** Gene Ontology (GO) Biological Process (BP Analysis.

**Supplementary Table S5.** Transcript Enrichment Analysis with KEGG.

**Supplementary Table S6.** Differentially expressed genes related to Wnt signaling. Raw data and GO molecular function and biological process data for animals treated with vehicle or C69 for 3 days post injury.

**Supplementary Table S7.** TRANSFAC data demonstrating transcription factors enriched among differentially expressed genes at all time points post injury in brain and spinal cord.

## Supplementary Videos

**Video S1.** 1A) Lamprey two days post spinal cord transaction shows paralysis caudal but not rostral to the lesion site. 1B) Recovery of swimming behavior demonstrated by swimming of an uninjured animal and an animal that is 9 weeks post transection. Initiation of swimming is prompted by tail pinch.

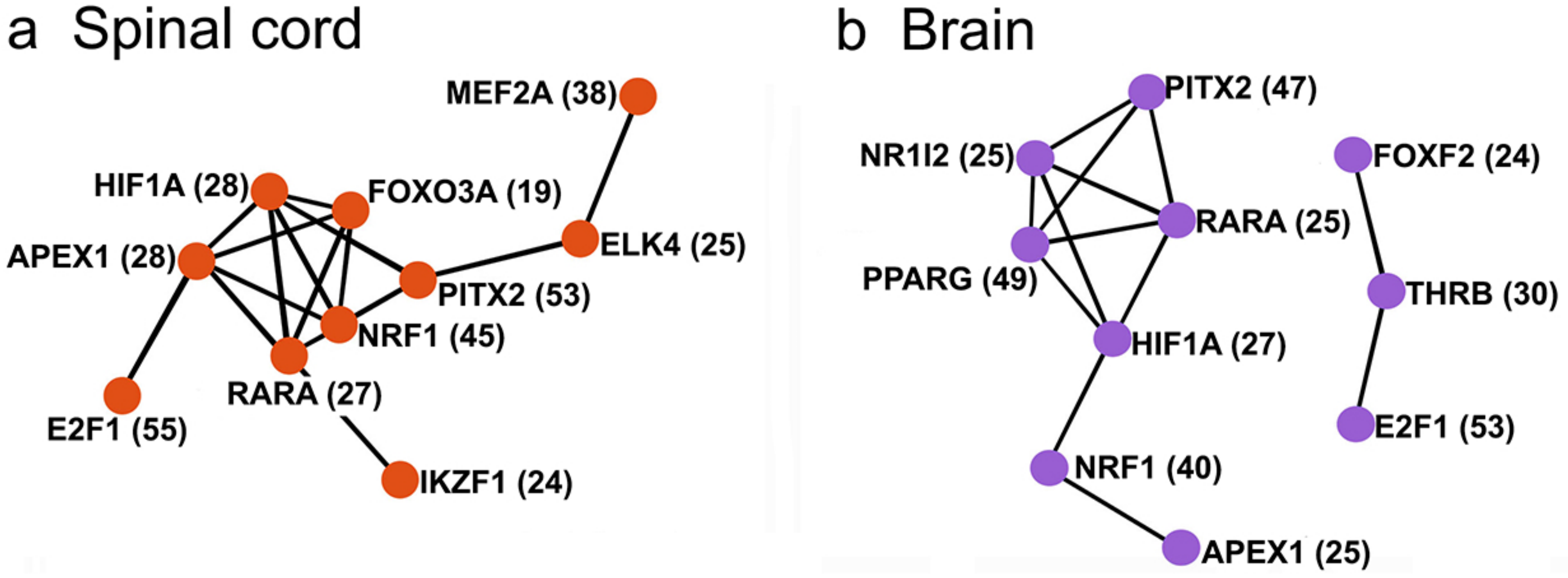


**Supplementary Figure S1**

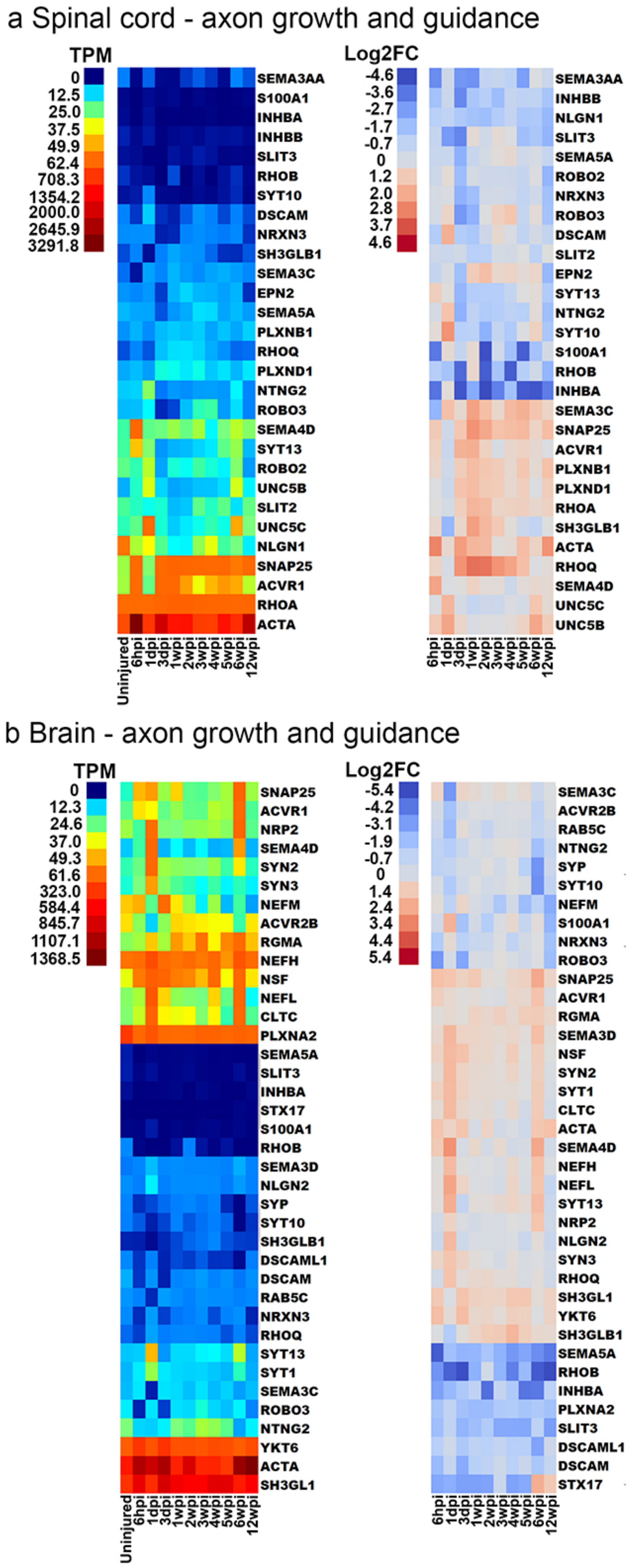


**Supplementary Figure S2**

a Spinal cord - extracellular matrix

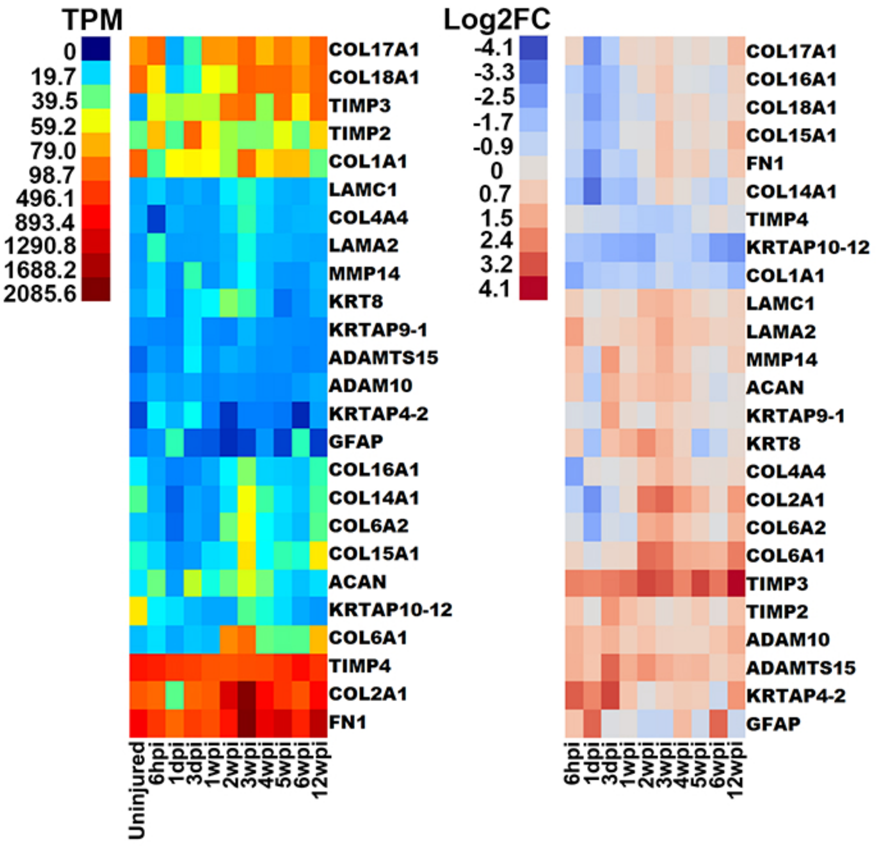


b Brain - extracellular matrix

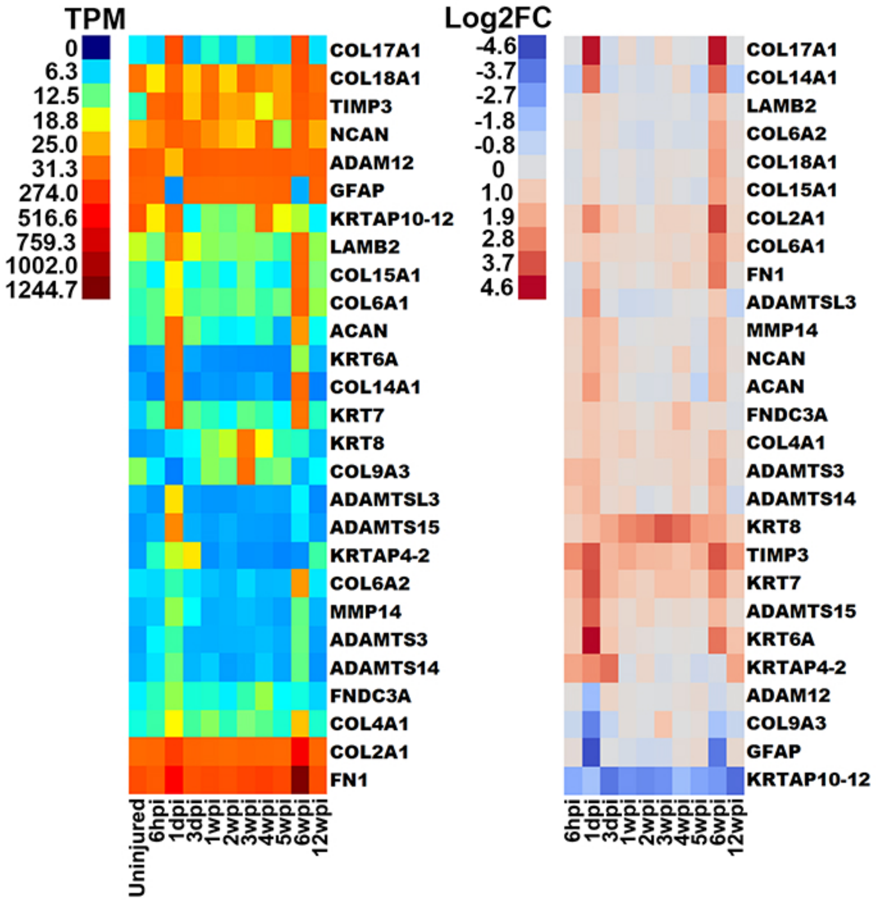


**Supplementary Figure S3**

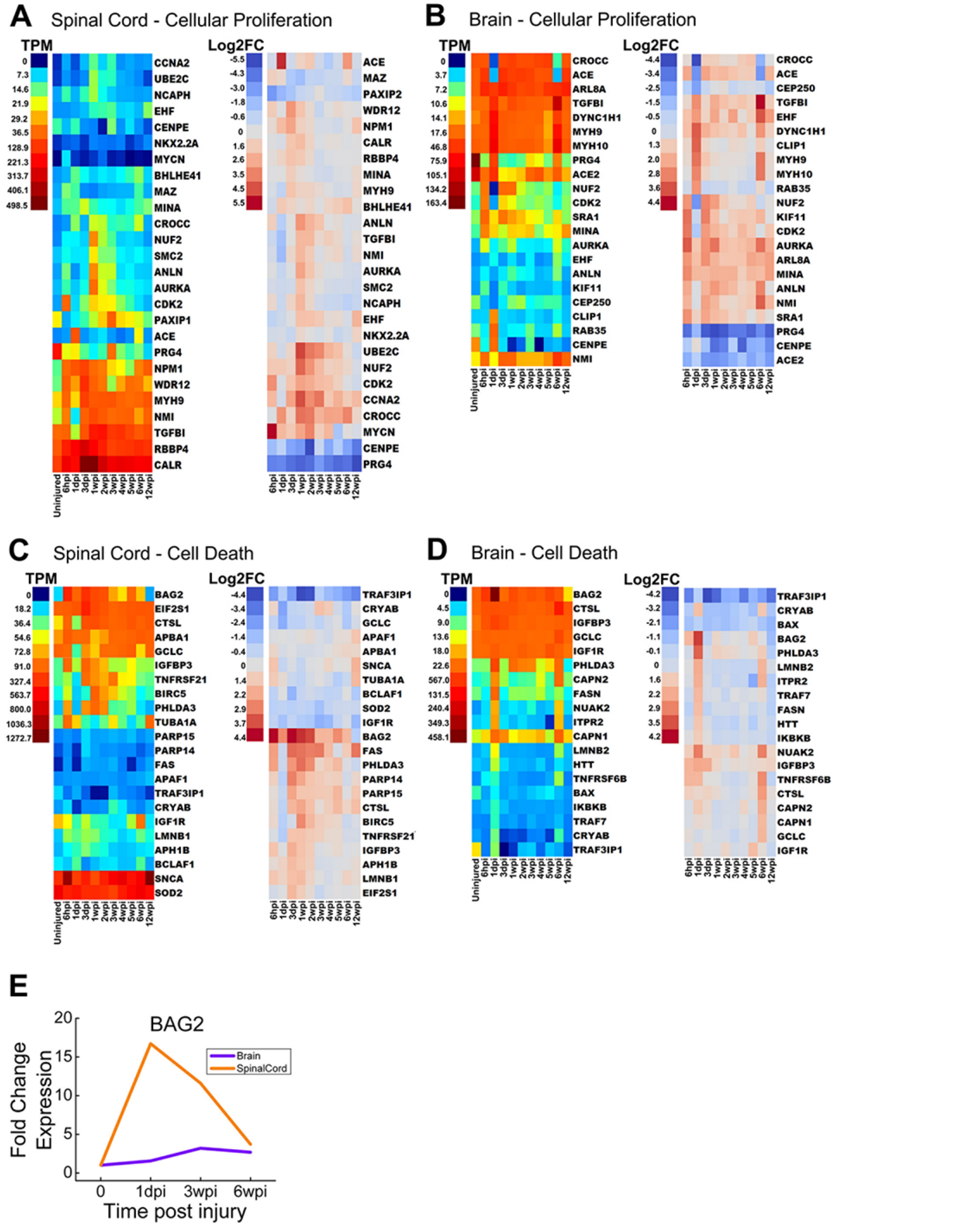


**Supplementary Figure S4**

a Spinal cord - ion channels

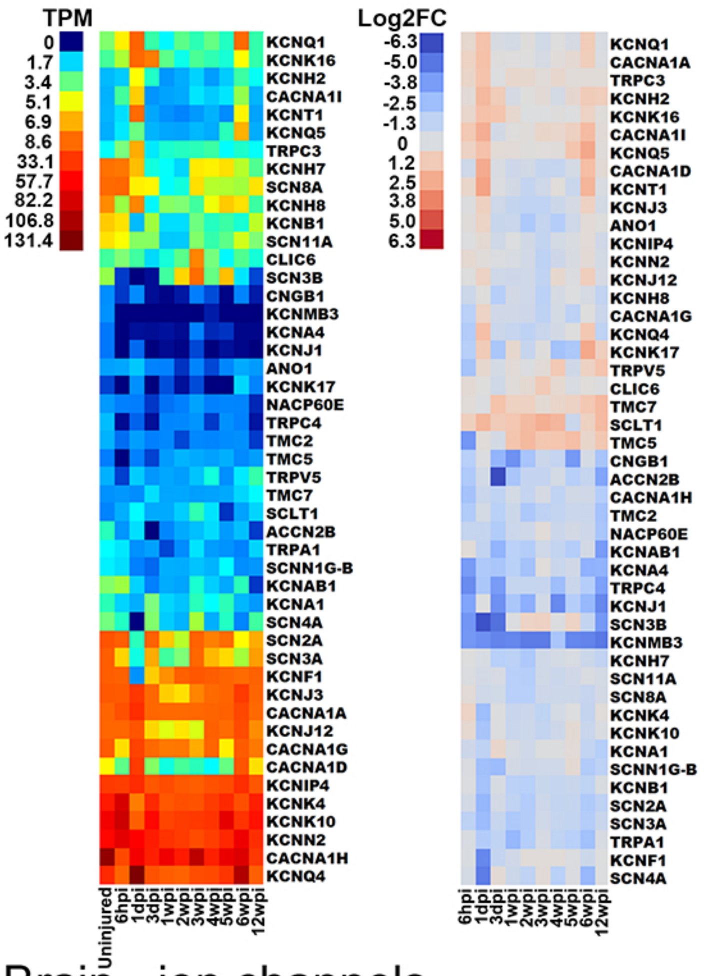


b Brain - ion channels

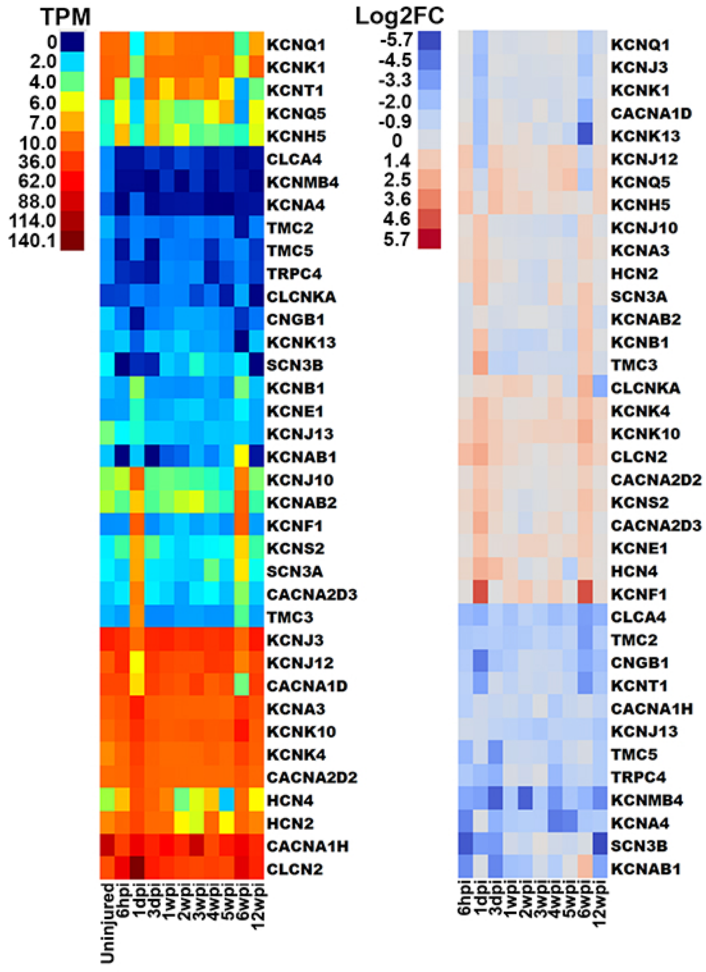


**Supplementary Figure S5**

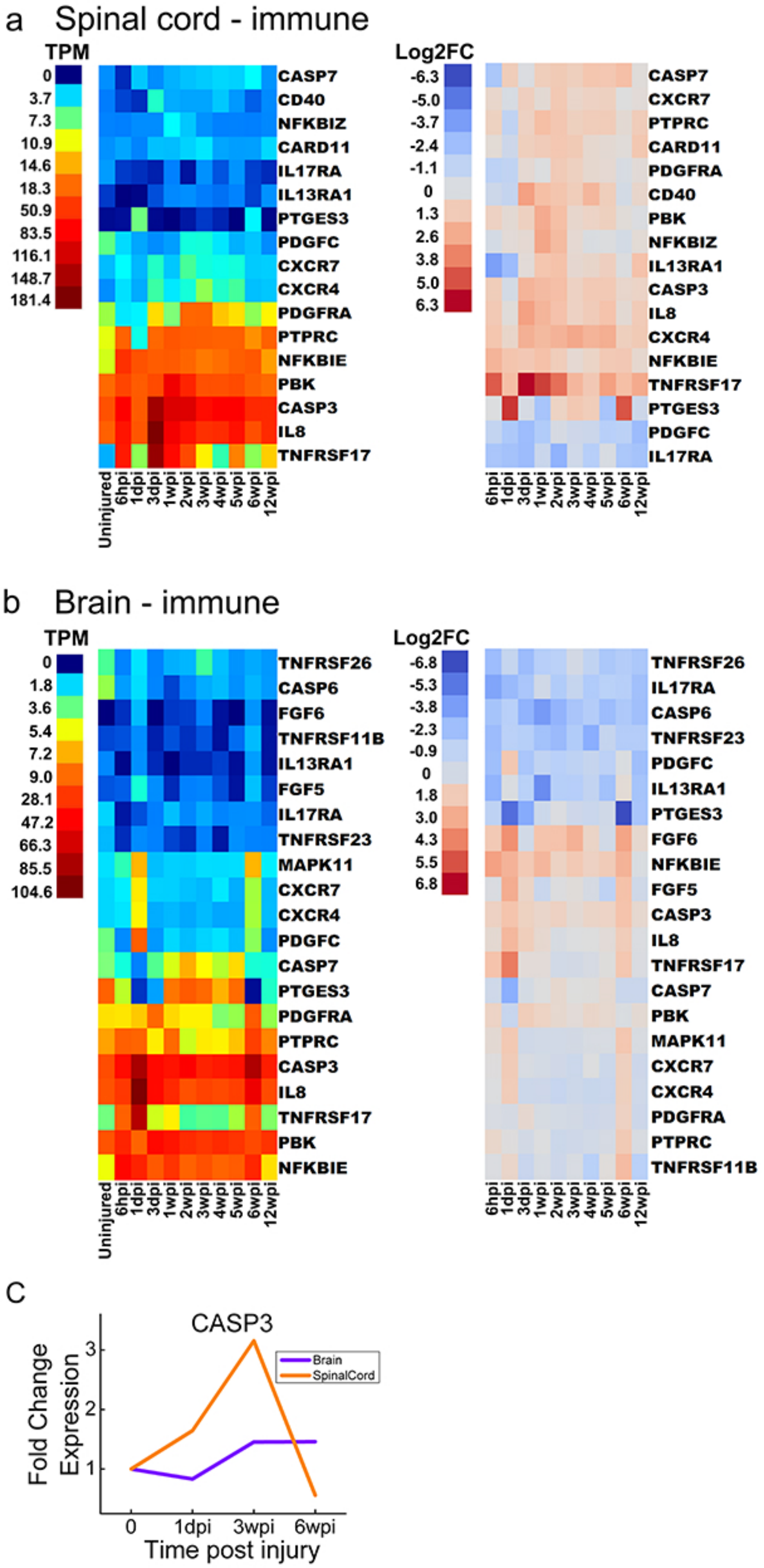


Supplementary Figure S6
